# Association of macular perfusion status with microvascular parameters up to the far periphery in diabetic retinopathy using multimodal imaging

**DOI:** 10.1186/s40942-020-00253-w

**Published:** 2020-11-04

**Authors:** Dorottya Hajdu, Aleksandra Sedova, Felix Datlinger, Julia Hafner, Irene Steiner, Katharina Kriechbaum, Christoph Scholda, Stefan Sacu, Ursula Schmidt-Erfurth, Andreas Pollreisz

**Affiliations:** 1 Department of Ophthalmology and Optometry, Vienna Clinical Trial Centre (VTC), Medical University of Vienna, Waehringer Guertel 18-20, E8i, 1090 Vienna, Austria; 2 Center for Medical Statistics, Informatics, and Intelligent Systems (CeMSIIS), Section for Medical Statistics, Medical University of Vienna, Vienna, Austria; 3 Christian Doppler Laboratory for Ophthalmic Image Analysis, Vienna Reading Center, Department of Ophthalmology and Optometry, Medical University of Vienna, Vienna, Austria

**Keywords:** Diabetic retinopathy, Retinal ischemia, SSOCTA, UWF, FA, DR severity

## Abstract

**Background:**

The aim of our study was to investigate a possible association between macular perfusion status and retinal ischemia and leakage up to far peripheral retinal areas in eyes with early to advanced stages of diabetic retinopathy (DR).

**Methods:**

In a retrospective, cross sectional analysis ultrawide field (UWF) color fundus photos (Optos, Optomap California) were graded for DR severity. Foveal avascular zone (FAZ) and vessel density from the superficial (SCP) and deep capillary plexus (DCP) were assessed on optical coherence tomography angiography (OCTA) scans (Topcon, DRI-OCT Triton). UWF angiography images were used to quantify leakage/ischemic index and number of microaneurysms (MA). Age, gender, disease duration, type of diabetes, HbA1C, hypertension, complications of diabetes and ocular history were recorded. Univariate mixed models and Spearman correlation analysis were used for statistical testing.

**Results:**

24 eyes of 17 laser-naive diabetic patients with different stages of DR were analyzed. The mean age was 59.56 ± 8.46 years and the mean disease duration 19.65 ± 12.25 years. No statistically significant associations between FAZ size, macular vessel density of SCP/DCP and peripheral retinal ischemia, leakage and MA number were demonstrated. Higher stages of DR were associated with ischemic index (estimate [95% CI]: 13.04 [1.5; 24.5], p = 0.033) and MA count (estimate [95% CI]: 43.7 [15.6; 71.8], p = 0.01), but no association with leakage index was observed. Only weak correlations between DR severity and anamnestic data were found.

**Conclusion:**

Retinal ischemic index and the amount of MAs assessed on UWFA up to peripheral areas are indicators of DR severity but not related to microvascular perfusion status in the macular region. Significance and timely sequence of macular vessel density in DR progression may need to be re-evaluated in future studies.

## Background

Diabetic retinopathy (DR) is the most common retinal microangiopathy and the leading cause of vision loss in the working age population with a global prevalence of 34.6% [1]. In recent years, DR research has focused on the investigation of ischemia in the peripheral retina.

In DR, the retinal microvasculature is affected by chronic hyperglycemia leading to blood flow abnormalities and retinal ischemia due to capillary dropout, which occurs mainly in the midperiphery of diabetic eyes [2, 3]. Histological studies show basement membrane disorder of the small vessels, loss of pericytes and obliteration of the precapillary arterioles associated with retinal capillary atrophy, leading to local capillary and arteriolar occlusions [4, 5]. These abnormalities cause circulatory disturbances primarily at the midperiphery due to distinct anatomical conditions of the intermediate capillary plexus (ICP), which gradually decreases from the fovea towards the periphery and becoming undetectable from 8 mm eccentricity potentially due to decreasing metabolic demand of the thinning retina [6]. The first occurrence of DR lesions have been reported to appear in the retinal periphery defined as the region outside the major vascular arcades and subdivided by the vortex vein ampulla into midperiphery and far periphery [3, 7, 8]. Recently, the presence of peripheral retinal lesions has been found to be associated with increased risk of DR progression [3, 9].

Ultrawide field (UWF) scanning laser ophthalmoscopy has revolutionized the imaging of the peripheral retina by allowing the acquisition of single capture images with a field of view of up to 200°, covering 80% of the retinal surface. This technology can be combined with fluorescein (FA) and indocyanine green angiography depicting the same field of view [10]. Previous studies analyzed angiographic features on UWFA, like ischemic index and leakage index, and found significant correlations with DR severity [11–13]. The panretinal evaluation of DR including angiographic features based on FA provides a more comprehensive picture of the disease and has a potential to predict outcomes and prognosis [10, 14]. Optical coherence tomography angiography (OCTA) offers noninvasive, detailed visualization of the retinal vasculature with numerous quantitative vessel parameters and makes the assessment of central diabetic features possible with the limitation of its inability to image the retinal periphery using commercially available systems in clinical routine [15, 16].

The aim of this study was to compare and correlate macular vascular parameters, and retinal ischemia and vessel leakage up to far peripheral retinal areas in eyes with different stages of DR. UWFA parameters, such as ischemic index, leakage index and microaneurysm (MA) number, as well as macular vascular parameters, namely foveal avascular zone (FAZ) and vessel densities of the superficial (SCP) and deep capillary plexus (DCP) as assessed by OCTA were evaluated.

## Methods

This study was performed according to the Declaration of Helsinki including current revisions and Good Clinical Practice (GCP) guidelines. The study protocol was approved by the Ethics Committee of the Medical University of Vienna. OCTA and UWFA images were acquired from August 2018 to June 2019 at the Department of Ophthalmology and Optometry of the Medical University of Vienna, Austria.

Inclusion criteria were: (1) diagnosis of DM, (2) clear ocular media, (3) absence of other concurrent ocular diseases, (4) absence of clinically significant macula edema, (5) no anti-VEGF injections within the last 8 weeks, (6) no previous steroid injections or implants, (7) no previous treatment with laser photocoagulation, (8) no proliferative retinopathy (PDR). Anamnestic data, such as type of diabetes, disease duration, HbA1c and systemic complications were documented based on the medical records provided by the primary care physician or specialist for internal medicine.

### SSOCT/-A analysis

Patients were examined with Topcon DRI-OCT Triton swept-source OCT/-A (Topcon, Japan) after pupil dilatation (Tropicamide, Mydriaticum) using 6 × 6 mm volumetric flow-scans centered on the fovea. The automated layer segmentation displays the different retinal vascular plexus. Manual correction of the segmentation based on the corresponding structural B-scan and image quality control was performed. The images with motion artifacts or unidentifiable dark areas were excluded from further analysis.

Vessel density of the SCP and DCP (expressed as percentage of vessel area with blood flow over total area measured) was analyzed within the standard ETDRS grid centered on the fovea using IMAGEnet 6 (version 1.22.1.14101) (Fig. 2). For further statistical analysis we used vessel density values of central, nasal, temporal, inferior and superior quadrants and the mean values of these areas excluding the central millimeter of the fovea. Manual measurement of FAZ was performed on the 6 × 6 mm OCTA using Image J (National Institute of Health, Bethesda, MD, USA).

### UWF and UWFA image analysis

**Fig. 1:**
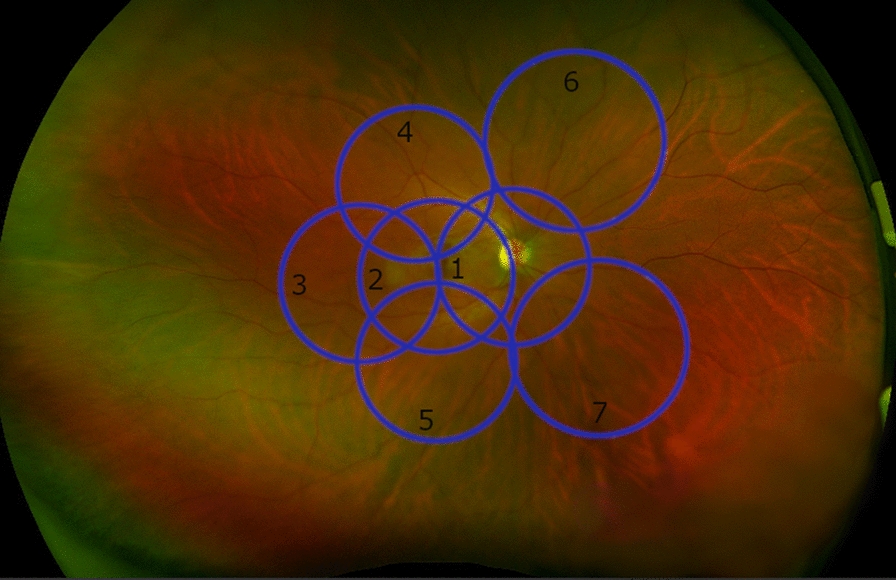
Optomap color fundus photo. Fundus photo from a right eye of a 30 years old healthy female with the superimposed seven round areas (blue), which correspond to the standard ETDRS 7-fields using the Optos Advanced System software. The black numbers showing the exact number of fields

**Fig. 2:**
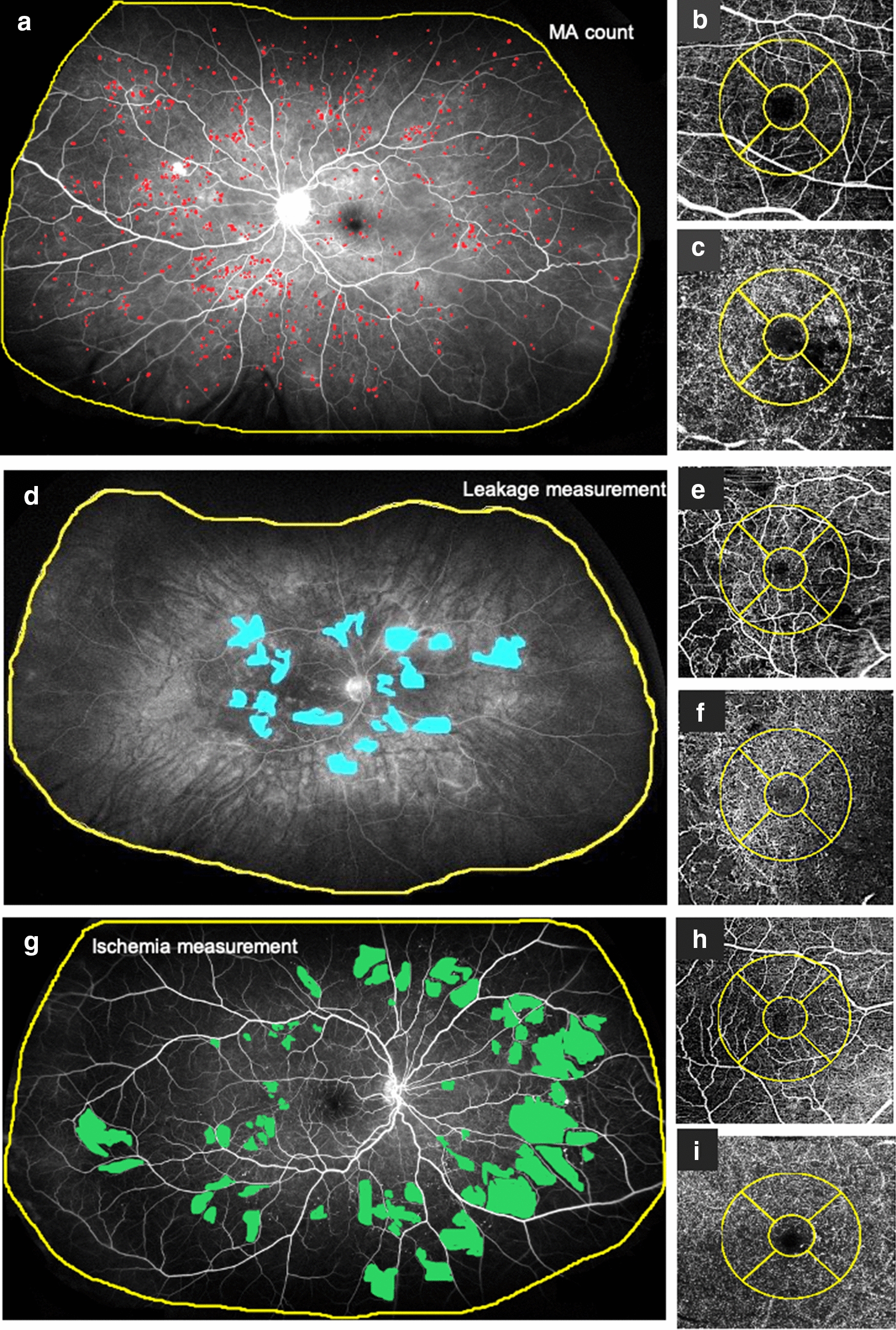
Ultrawide field angiography (UWFA) and optical coherence tomography angiography (OCTA) images of three diabetic patients. Patient 1 **a**–**c**: 60 years old female patient with severe DR. Patient 2 **d**–**f**: 49 years old female patient with moderate DR. Patient 3 **g**–**i**: 52 years old male patient with moderate DR. OCTA images shows the superficial (SCP) (**b**, **e**, **h**) and deep (DCP) (**c**, **f**, **i**) capillary plexus with the ETDRS Grid (yellow circle). The yellow line on the UWFA images (**a**, **d**, **g**) indicates the region of interest (ROI). **a** Microaneurysm (MA) analysis using an early phase UWFA image. Red dots indicate the MA-s. **b** OCTA image of the SCP of patient 1. **c** OCTA image of the DCP of patient 1. **d** Leakage analysis using a late phase UWFA image. Blue areas correspond to the leakage areas. **e** OCTA image of the SCP of patient 2. **f** OCTA image of the DCP of patient 2. **g** Ischemia analysis in an early phase UWFA image. The green field show ischemic areas. **h** OCTA image of the SCP of patient 3. **i** OCTA image of the DCP of patient 3

Patients underwent routine UWF color imaging using the Optos system (Optomap, California) with a 21-inch and 1920 × 1080 pixel resolution display used for grading. After quality control the non-gradable images were excluded and the same investigator graded one 200° UWF image of each of the patients for the presence and severity of DR. We superimposed seven round areas on UWF images, which correspond to the standard ETDRS 7-fields using the Optos Advance device software (version 4.231.94248) (Fig. 1). Grading was performed according to EDTRS and diabetic retinopathy severity score (DRSS) criteria from the ETDRS 7-fields [17, 18]. Based on the classification, the following nomenclature was used: no, mild, moderate, and severe non-proliferative diabetic retinopathy (NPDR). UWFA images were evaluated to identify the number of MAs, ischemic index and leakage index, according to the method of a previous study from Ehlers et al. [13] The region of interest (ROI) was defined as a gradable area without artifacts and eyelashes (see Fig. 2).

### Statistical methods

Data were analyzed with univariate mixed models with patient as a random factor. An observation was excluded from the model if the following criterion was met: restricted likelihood distance RLD > 2 and Cook’s distance > 4/n and |CovRatio—1|> 3*p/n, where n is the number of eyes and p is the number of parameters in the model [19, 20]. The estimate of the slope β1 with 95% confidence intervals and the p-value (H0: β1 = 0) are reported. Mean vessel density was defined as the mean of inferior, superior, temporal and nasal region. The correlation between age, HbA1c, and the duration of diabetes with DR severity was analyzed by Spearman correlations. As the requirement of independent observations is not met, only the Spearman correlation coefficients but no p-values or confidence intervals are reported. Statistical analyses were conducted with SAS 9.4. and R 3.6.2. P-values smaller than 0.05 were considered statistically significant. Due to the exploratory character of the study, we did not adjust for multiple testing. Hence the interpretation of the results is done in a descriptive manner.

## Results

A total of 48 eyes from 33 consecutive diabetic patients presenting at the Department of Ophthalmology at the Medical University of Vienna between August 2018 and June 2019 met the inclusion criteria including same day OCTA and UWFA imaging and were analyzed in this study. Twenty-three eyes were excluded due to insufficient OCTA image quality preventing appropriate analysis and interpretation. Out of the remaining 24 eyes of 18 patients (4 females) 19 eyes were type II and 5 eyes type I diabetic eyes. The mean age (± SD) was 59.56 ± 8.46 years and the mean disease duration 19.65 ± 12.25 years. Mean HbA1C levels were 7.46 ± 1.31% with data from 4 patients not available. Nephropathy was present in 2 while polyneuropathy in 4 patients. Three patients had a history of myocardial infarction or stroke. Visual acuity was 0.8 ± 0.24 (Snellen 20/25). Ten eyes received anti-VEGF treatment (Aflibercept) (median (minimum; maximum) 4 (1; 9) injections) with the last injection administered at least 8 weeks before study inclusion. Seven eyes were pseudophakic and seventeen phakic. A complete listing of the anamnestic data is given in Table 1.

**Table 1:** Patient characteristics and anamnestic data

Clinical characteristics	
Female sex, n patient (%)	4 (22)
Age, mean ± SD in years	59.56 ± 8.46
Disease duration, mean ± SD in years	19.65 ± 12.25
Diabetes Type n patient (%)	
Type I	4 (22)
Type II	14 (78)
HbA1c, mean ± SD in % *	7.46 ± 1.31%
History of hypertension, n patient (%)*	7 (39)
History of nephropathy, n patient (%)*	2 (11)
History of polyneuropathy, n patient (%)*	4 (22)
History of heart attack/ stroke, n patient (%)	3 (17)
Visual acuity mean ± SD in Snellen decimal	0.8 ± 0.24
Lens status, n eyes (%)	
Phakic	17 (71)
Pseudophakic	7 (29)
DR severity, n eyes (%)	
No DR	2 (8)
Mild DR	9 (38)
Moderate DR	11 (46)
Severe DR	2 (8)

*Based on the medical records provided by the primary care physician or specialist for internal medicine

*HbA1C* hemoglobin A1C, *DR* diabetic retinopathy, *SD* standard deviation

DR severity was graded on ETDRS 7-fields superimposed on Optomap images. Two eyes were classified as having no DR, whereas 9 had mild NPDR, 11 moderate NPDR and 2 severe NPDR. Two eyes were graded as no DR according to the ETDRS criteria as this grading system is based on the presence of microaneurysms and retinal hemorrhages. In these two eyes, no such features were found, yet, pronounced areas of capillary dropout were detected on OCTA and UWFA and therefore included in the analysis.

In six patients both eyes were included with two patients presenting with different stages of DR (mild and moderate). Three patients had different pre-treatments, namely one patient was phakic in one eye and pseudophakic in the other, while two patients received anti-VEGF injections in one eye and no treatment in the other eye.

Vessel density of the SCP and DCP and FAZ were evaluated on 6 × 6 mm OCTA images. The mean FAZ area was 0.25 ± 0.155 mm^2^, mean vessel density of the SCP 44.43 ± 1.87% and mean vessel density of the DCP 46.52 ± 2.33%.

Early mid-phase (up to 2 min after fluorescein injection) UWFA images were used for MA counting and ischemia assessment. Ischemia was defined as capillary nonperfusion and ischemic index as the percentage area of ischemia within the ROI. The mean MA number in eyes with no DR was 7.5 ± 3.5, with mild NPDR 74 ± 65.2, with moderate NPDR 235.7 ± 160.4 and with severe NPDR 154 ± 1.4. The average value of the ischemic index was 10.2 ± 7.13% in eyes with no DR, 27.81 ± 22.7% with mild DR, 43.62 ± 9.9% with moderate NPDR and 44.78 ± 13.3% with severe NPDR. Late-phase images (between 2–6 min) were evaluated for manually leakage analysis. For further statistical analysis leakage index was used as the percentage area of leakage within the ROI. Leakage index revealed a value of 0.9 ± 0.78% in eyes with no DR, 9.41 ± 8.48% with mild NPDR, and 10.24 ± 9.64% with moderate NPDR and 28.88 ± 18.77% with severe NPDR.

The association between ischemic index, leakage index and vessel density and FAZ area was calculated to evaluate the relationship between central vascular parameters, retinal ischemia and vessel leakage. We found no statistically significant association between central parameters (FAZ and vessel density) assessed by OCTA and retinal ischemia and leakage assessed by UWFA in this study cohort. No statistically significant effect of ischemic index on vessel density of SCP and DCP was found. Also, there was no association between leakage index and vessel density of both plexuses. Ischemic index and leakage index were not associated with FAZ area (see Table 2).

**Table 2:** Results of the univariate linear mixed models with ischemic index or leakage index, respectively, as independent variable

Dependent variable	Ischemic index		Leakage index	
	Estimate [95% CI]	p-value	Estimate [95% CI]	p-value
FAZ area	−0.0001 [−0.0026, 0.0029]*	0.91	−0.0015 [−0.0059, 0.0029]*	0.41
VD central SCP	−0.044 [−0.18, 0.092]	0.45	−0.0035 [−0.24, 0.24]	0.99
VD superior SCP	−0.022 [−0.11, 0.062]	0.54	−0.012 [−0.16, 0.13]	0.84
VD nasal SCP	−0.017 [−0.11, 0.073]	0.65	−0.037 [−0.19, 0.12]	0.57
VD inferior SCP	−0.013 [−0.093, 0.067]	0.70	−0.049 [−0.18, 0.084]	0.39
VD temporal SCP	−0.041 [−0.11, 0.029]	0.19	−0.0088 [−0.13, 0.11]	0.86
VD mean SCP	−0.024 [−0.072, 0.025]	0.27	−0.025 [−0.11, 0.059]	0.48
VD central DCP	−0.062 [−0.18, 0.058]	0.24	−0.0065 [−0.22, 0.21]	0.94
VD superior DCP	−0.032 [−0.12, 0.059]	0.41	−0.091 [−0.32, 0.14]*	0.33
VD nasal DCP	−0.057 [−0.17, 0.055]	0.25	−0.15 [−0.34, 0.033]	0.087
VD inferior DCP	−0.026 [−0.12, 0.068]	0.51	−0.098 [−0.25, 0.056]	0.16
VD temporal DCP	−0.037 [−0.10, 0.029]	0.21	0.015 [−0.10, 0.13]	0.75
VD mean DCP	−0.044 [−0.11, 0.019]	0.13	−0.090 [−0.19, 0.011]	0.071

* Indicates one influential observation excluded from analyses.

*FAZ* foveal avasculare zone, *VD* vessel density, *SCP* superficial capillary plexus, *DCP* deep capillary plexus

The effect of DR severity on ischemic index, leakage index, FAZ, vessel density, and visual acuity was also examined, and we found a statistically significant association between ischemic index and DR severity. With increasing DR severity, ischemic index increased (estimate [95% CI]: 13.04 [1.54; 24.54], p = 0.033). A statistically significant effect of DR severity on the amount of MAs was also demonstrated (estimate [95% CI]: 43.67 [15.6; 71.8], p = 0.01, one influential observation removed). The effect of DR severity on leakage index was not found to be statistically significant (estimate [95% CI]: 5.86 [−1.2; 12.97], p = 0.087). No statistically significant effect of DR severity on FAZ area (p = 0.61, one influential observation removed), and vessel density of SCP (central: p = 0.25, mean: p = 0.41), DCP (central: p = 0.12, mean: p = 0.98), and visual acuity (p = 0.73, one influential observation removed),) was found. A statistically significant effect of central and mean vessel density of SCP and DCP on visual acuity was found, whereas the effect of ischemic index, leakage index, FAZ area and central vessel density of the DCP was statistically not significant (see Table 3).

**Table 3:** Results of the univariate mixed models with visual acuity as dependent variable

Independent variable	Estimate [95% CI]	p-value
Ischemic index	−0.0025 [−0.0097; 0.0047]	0.41
Leakage index	−0.0027 [−0.013; 0.007]*	0.49
FAZ area	−0.188 [−0.989; 0.614]*	0.55
VD central SCP	−0.024 [−0.041; −0.0065]*	0.019
VD mean SCP	0.067 [0.014; 0.12]*	0.024
VD central DCP	−0.018 [−0.042; 0.0066]*	0.11
VD mean DCP	0.042 [0.0024; 0.082]*	0.042

* Indicates one influential observation excluded from analyses.

*FAZ* foveal avasculare zone, *VD* vessel density, *SCP* superficial capillary plexus, *DCP* deep capillary plexus

For subsequent analysis the correlation between age, HbA1c and disease duration with DR severity was analyzed. We observed poor correlation between age and disease duration with DR severity (age: rs = −0.02, n = 24 eyes; disease duration: rs = 0.03, n = 22 eyes), and a weak negative correlation between DR severity and HbA1c (rs = −0.38, n = 19 eyes).

## Discussion

The present study investigated a possible association of retinal vessel densities in the posterior pole with retinal ischemia and vessel leakage up to far peripheral retinal areas in patients with early to advanced DR using a multimodal imaging approach including OCTA and UWFA. Our results revealed that unlike retinal vessel densities in the central posterior pole, angiographic findings of ischemia and leakage up to the retinal periphery were the strongest associated features with DR severity. Higher stages of DR were significantly associated with MA number and ischemic index up to the far periphery, but no association with leakage index was observed.

Retinal ischemia is known to induce the release of VEGF in eyes presenting more nonperfused areas with a higher probability of developing DME [14, 21]. The detection and monitoring of peripheral retinal ischemia plays an important role in DR management as patients with large nonperfused areas on UWFA may necessitate closer monitoring and earlier follow-up than those with no evidence of retinal ischemia [14]. Niki et al. evaluated 152 diabetic eyes with nonproliferative DR using composite widefield fluorescein angiography images and reported that nonperfused areas are located primarily in the midperipheral retina [3].

Angiographic imaging techniques depicting the retinal periphery by montaging several images result in a number of disadvantages including the need of mydriasis, patient cooperation and technical skills by the photographer [22, 23]. Non-mydriatic UWF imaging, as employed in our study, has expanded the field of view of up to 200°, covering 80% of the retinal surface in one single image [10]. Several studies highlighted the importance of the peripheral retina in DR and found that diabetic lesions in the retinal periphery are associated with increased risk of DR progression [10, 12, 14].

The detection of lesions in the mid- and far peripheries using UWF fundus photography has yielded higher levels of DR in 9–39% of eyes [10, 24, 25].

Wessels et al. showed that UWFA can detect 3.9 times more nonperfused areas compared with the 7 standard fields [9]. However, UWFA has limited resolution compared with OCTA, and leakage may obscure ischemic areas [22].

Rabiolo et al. measured nonperfusion and found a significant correlation with DR severity [11]. Leakage and ischemic index, as well as the amount of MAs are associated with DR severity as reported by Ehlers et al. [13]. Our results confirm these reports by showing that ischemic index and MA number were associated with DR severity. However, an association between DR severity and leakage index did not reach a significant level. This may be explained by the fact that we did not include proliferative DR patients who potentially present a larger leakage area due to active neovascularization.

Further, studies investigated the relationship between FAZ and ischemic index assessed on UWFA images and reported positive correlations between these parameters [11, 26]. Or et al. analyzed 82 diabetic eyes with OCTA and UWFA and reported a positive association between FAZ and peripheral ischemia. However, macular vessel densities did not exhibit a linear relationship with peripheral capillary nonperfusion as assessed on UWFA images [27]. It should be noted that these studies included eyes with DME potentially affecting the accurate identification of nonperfused areas in the macula. Our results do not correspond with the previously reported findings as we could not show any association between macular nonperfusion (FAZ, vessel density) and retinal ischemic and leakage indices including the far periphery.

The effect of intravitreal anti-VEGF injections on retinal vessels was investigated in numerous previous studies but the reports are controversial. DR severity improvement based on the ETDRS and DRSS grading was demonstrated 1 month after anti-VEGF injection with decreased vascular leakage observed on UWFA images [33, 34]. The extent of retinal nonperfusion assessed by fluorescein angiography was found to be decreased 1 month after treatment [35] and reported unchanged in other studies after 6–12 month [33, 35]. No significant differences were reported in parafoveal vascular densities, FAZ, vascular caliber changes or reperfusion before and up to 12 month after anti-VEGF therapy [28–32]. However, other authors demonstrated transient changes in vascular parameters, namely FAZ enlargement and retinal arteriolar vasoconstriction 4–6 weeks after anti-VEGF injections [36, 37]. In order to avoid potential effects of the drug on blood vessels we set our inclusion criteria based on these results and only included patients who had not received any injection in the previous 8 weeks.

Some limitations of this study should be recognized. First of all, it is a retrospective study and has a relatively small sample size with DR stages not equally balanced. Secondly, leakage on UWFA images may cover ischemic areas, which we addressed by evaluating early phase images. Thirdly, 10 eyes of 8 patients enrolled were treated with anti-VEGF injections before. Although the time point of last injection was set to a minimum of 2 months, it cannot be ruled out that the pretreatment had an effect on our results. Furthermore, anti-VEGF may potentially influence macular ischemia and peripheral ischemia differently.

## Conclusions

In conclusion, this study shows that ischemic index and number of MAs as assessed on UWFA are associated with DR severity but not with macular vessel densities of superficial and deep capillary plexuses evaluated with OCTA. In order to fully confirm these assumptions larger, prospective studies with higher resolution of peripheral retinal microvasculature are needed.

## Data Availability

The datasets used and/or analyzed during the current study are available from the corresponding author on reasonable request.

## Abbreviations

DCP: Deep capillary plexus
DME: Diabetic macular edema
DR: Diabetic retinopathy
ETDRS: Early Treatment Diabetic Retinopathy Study
FA: Fluorescein angiography
FAZ: Foveal avascular zone
GCP: Good Clinical Practice
HbA1C: Hemoglobin A1c
ICP: Intermediate capillary plexus
MA: Microaneurysm
NPDR: Non-proliferative diabetic retinopathy
OCT: Optical coherence tomography
OCTA: Optical coherence tomography angiography
OCTARA: OCTA Ratio Analysis
PDR: Proliferative diabetic retinopathy
ROI: Region of interest
RLD: Restricted likelihood distance
SCP: Superficial capillary plexus
SD: Standard deviation
SSOCT: Swept-source OCT
UWF: Ultrawide field
UWFA: Ultrawide field fluorescein angiography
VEGF: Vascular endothelial growth factor
